# Uncovering impaired mitochondrial and lysosomal function in adipose-derived stem cells from obese individuals with altered biological activity

**DOI:** 10.1186/s13287-023-03625-9

**Published:** 2024-01-08

**Authors:** Bo Wang, Ge Zhang, Yuwen Hu, Ali Mohsin, Zhimin Chen, Weijie Hao, Zhanxia Li, Wei-Qiang Gao, Meijin Guo, Huiming Xu

**Affiliations:** 1 State Key Laboratory of Bioreactor Engineering, East China University of Science and Technology, 130 Meilong Rd., P.O. Box 329#, Shanghai, 200237 People’s Republic of China; 2 State Key Laboratory of Oncogenes and Related Genes, and Renji-MedX Clinical Stem Cell Research Center RenJi Hospital, Shanghai Jiao Tong University School of Medicine, Shanghai, 200127 People’s Republic of China; 3 Department of Respiratory Medicine, Shanghai Sixth People’s Hospital Affiliated to Shanghai Jiao Tong University School of Medicine, 600 Yishan Road, Xuhui District, Shanghai, 200235 People’s Republic of China; 4 Med-X Research Institute and School of Biomedical Engineering, Shanghai Jiao Tong University, Shanghai, 200030 People’s Republic of China

**Keywords:** Adipose-derived stem cells, Obesity, Mitochondrial dysfunction, Lysosomal dysfunction

## Abstract

**Background:**

Adipose-derived stem cells (ADSCs) have been extensively used in preclinical and clinical trials for treating various diseases. However, the differences between ADSCs from lean individuals (L-ADSCs) and those from obese individuals (O-ADSCs) have not been thoroughly investigated, particularly regarding their mitochondrial and lysosomal functions. Therefore, this study aims to evaluate the differences between L-ADSCs and O-ADSCs in terms of cell biological activity, mitochondria, and lysosomes.

**Methods:**

We first isolated and cultured L-ADSCs and O-ADSCs. We then compared the differences between the two groups in terms of biological activity, including cell proliferation, differentiation potential, and their effect on the polarization of macrophages. Additionally, we observed the mitochondrial and lysosomal morphology of ADSCs using an electronic microscope, MitoTracker Red, and lysotracker Red dyes. We assessed mitochondrial function by examining mitochondrial membrane potential and membrane fluidity, antioxidative ability, and cell energy metabolism. Lysosomal function was evaluated by measuring autophagy and phagocytosis. Finally, we performed transcriptome analysis of the ADSCs using RNA sequencing.

**Results:**

The biological activities of O-ADSCs were decreased, including cell immunophenotypic profiles, cell proliferation, and differentiation potential. Furthermore, compared to L-ADSCs, O-ADSCs promoted M1-type macrophage polarization and inhibited M2-type macrophage polarization. Additionally, the mitochondrial morphology of O-ADSCs was altered, with the size of the cells becoming smaller and mitochondrial fragments increasing. O-ADSCs also exhibited decreased mitochondrial membrane potential and membrane fluidity, antioxidative ability, and energy metabolism. With respect to lysosomes, O-ADSCs contained ungraded materials in their lysosomes, enhanced lysosomal permeability, and reduced autophagy and phagocytosis ability. RNA sequence analysis indicated that the signalling pathways related to cell senescence, cancer, and inflammation were upregulated, whereas the signalling pathways associated with stemness, cell differentiation, metabolism, and response to stress and stimuli were downregulated.

**Conclusions:**

This study indicates that ADSCs from individuals (BMI > 30 kg/m^2^) exhibit impaired mitochondrial and lysosomal function with decreased biological activity.

**Supplementary Information:**

The online version contains supplementary material available at 10.1186/s13287-023-03625-9.

## Background

Currently, adipose-derived stem cells (ADSCs) are widely used in preclinical and clinical trials for treating various diseases [1]. It has been shown that ADSCs from lean individuals (L-ADSCs) and obese individuals (O-ADSCs) have different biological properties. O-ADSCs exhibit reduced proliferation ability, differentiation potential, and immunomodulatory ability in vitro compared to those from lean donors (L-ADSC) [2–6]. In a murine experimental autoimmune encephalomyelitis (EAE) model, O-ADSCs fail to inhibit the inflammation in the central nervous system and alleviate the symptoms [7]. In addition, previous studies indicate that ADSCs or adipose progenitor can be key regulators of macrophage recruitment and activation in white adipose tissue (WAT) and the metabolic inflammation established during obesity [3, 8, 9]. On the other hand, obesity is shown to alter the immune properties of ADSCs [2]. Moreover, weight loss can partially restore cell proliferation, viability, and regenerate properties of ADSCs from formerly obese mice [10]. The studies suggested that obesity alters the biological properties of ADSCs while ADSCs also contribute to the establishment and maintenance of the inflammation state in obesity. Therefore, comprehensive understanding the properties of L-ADSCs and O-ADSCs can be critical for developing new therapeutic strategies to treat obesity and its related complications.

Obesity has been demonstrated to be related to mitochondrial dysfunction [11]. Mitochondria are organelles responsible for energy metabolism and ATP production. Apart from ATP synthesis, they are involved in processes such as β-oxidation of fatty acid, calcium homoeostasis, iron transport, and the generation and elimination of reactive oxygen species (ROS). Additionally, mitochondria also play a role in apoptosis, ferroptosis, and inflammasome activation [12–15]. Mitochondrial homeostasis or mitochondrial dynamics requires a balance of mitochondrial fission and fusion, which control the mitochondrial morphology, quality, quantity, and inheritance [16]. Excess nutrient consumption can lead to high concentration of free fatty acids, hyperglycemia, increase ROS production and cause adipocyte mitochondrial dysfunction [17]. It is reported that the downregulation of mitochondrial biogenesis in obesity is associated with insulin resistance and low-grade inflammation [11]. Therefore, these studies indicate that the mitochondrial dysfunction in obesity is associated with inflammation, insulin resistance, and hyperglycemia. Regarding the relationship of cell activity and mitochondrial function, our previous study shows that mesenchymal stem cells (MSCs) after long-time culture *in vitro* exhibit senescence phenotype, reduced biological activity and impaired mitochondrial morphological and function [18]. In hematopoietic stem cells (HSCs), the integrity of mitochondrial respiratory chain is essential for HSC differentiation and maintenance of adult HSC quiescence [19]. These reports reveal that mitochondria function is closely associated to the activity of stem cells. However, the mitochondrial morphology and function of O-ADSCs have been less investigated.

In addition to mitochondrial dysfunction, lysosomal dysfunction has also been reported in obesity [20]. Lysosomes, being vital organelles in degrading various extracellular and cellular components. It is also play important roles in cell signaling transduction, cell homeostasis, metabolism, membrane repair, development and aging [21–23]. Autophagy refers to the lysosomal degradation and recycling of intracellular components [24]. Autophagy can remove dysfunctional mitochondria (known as mitophagy) to limit ROS production and control mitochondrial homeostasis. The perturbation of autophagy can impact the development of obesity and diabetes [20]. Moreover, autophagy interplays with inflammation. Autophagy has an important role in regulating inflammation, which in turn, autophagy can be activated by inflammatory components and inflammatory signaling-related elements [25]. These data reveal that lysosome dysfunction is associated with obesity and inflammation. In addition, amount of evidence indicates that lysosomes are required to maintain stem cell activity and function and influence stem cell fate [26–28]. In quiescent neural stem cells, impaired lysosome function causes more accumulation of protein aggregates and decreases the activation of quiescent neural stem cells [26]. Our previous study found that MSCs after long-time culture exhibit reduced autophagy and lysosomal dysfunction [18]. In hematopoietic stem cells, lysosomes are asymmetrically distributed in daughter cells, affecting the fate of recipient cells [29]. The data suggest lysosomes play a key role in the maintenance of stem cell activity. However, the lysosomal morphology and function of O-ADSCs is less investigated.

In the present study, we will first compare the biological activity of L-ADSCs and O-ADSCs and focus on investigating the changes in mitochondrial and lysosomal function in O-ADSCs. Our findings suggest that the biological activity is reduced in O-ADSCs, and the function of mitochondria and lysosome is impaired.

## Materials and methods

### Isolation and culture of ADSCs from lean and obesity donors

L-ADSCs and O-ADSCs were isolated from the liposuction specimens of lean donors (BMI < 25 kg/m^2^) and donors with obesity (BMI ≥ 30 kg/m^2^), respectively. *N* ≥ 3 is for each group. Moreover, the individuals have no other serious diseases, such as diabetes, cardiovascular disease, liver, kidney diseases. Prior to the collection of samples, the women provided informed consent, and the collection and use of the specimens were approved by the Human Research Ethics Committee of RenJi Hospital, School of Medicine, Shanghai Jiao Tong University. ADSCs were isolated according to the previously described protocol [30]. Briefly, the subcutaneous white adipose tissue was washed with HBSS (ThermoFisher Scientific) and digested with type I collagenase (0.1 mg/ml, Sigma-Aldrich) in a water bath shaker at 37 °C for 30 min. The cell pellet was then resuspended in DMEM/F12 (ThermoFisher Scientific) with 5% UltroGro (Helios Bioscience) and 1% penicillin/streptomycin (P/S). Finally, the ADSCs were passaged at 90% confluence, and ADSCs at passage 3 (P3)–P4 were used in the following experiments.

### Cell proliferation assay of ADSCs

Cells were seeded into a 96-well plate (2000 cells/well), and cell proliferation was measured by CCK-8 kit (Dojindo) from day 1 to day 3.

### Flow cytometry

Cells were digested with trypsin and washed with cold PBS, and then stained with isotype IgG or the following monoclonal antibodies: CD106-PE, CD36-PE, CD90-FITC, CD105-APC, CD73-FITC, CD34-PerCP, CD45-FITC, CD127-FITC, CD31-FITC (all from eBioscience). After being washed by PBS, the labeled cells were resuspended in PBS supplemented with 0.2% FBS and at least 105 events were acquired using BD Accuri™ C6 Flow cytometer (BD, NJ, USA).

### β-Galactosidase staining

ADSCs were cultured in 12-well plates upon 90% confluence and then stained with senescence β-galactosidase (β-gal) assay Kit (Beyotime) according to the manufacturer’s instruction.

### Cell apoptosis assay

Cells were collected and stained using the Annexin V-APC/PI apoptosis detection kit (BioLegend). Then, the cells were analyzed by BD Accuri™ C6 Flow cytometer.

### Adipogenic, osteogenic and chondrogenic differentiation

To evaluate the adipogenic and osteogenic potential of ADSCs, cells at 90% confluence were cultured with adipogenic or osteogenic differentiation medium (STEMCELL Technologies) for 10–15 days. The differentiation medium was replaced every 2 days. Adipogenic and osteogenic differentiation were assessed by Oil Red O staining and alizarin red staining, respectively.

For chondrogenic differentiation, a previously described protocol was followed [31]. Briefly, ADSCs were cultured in a normal cell growth medium at 37 °C with 5% CO_2_ for 24 h, then the cells were cultured with chondrogenic differentiation medium (STEMCELL Technologies), which was replaced every 3 days. After 28–30 days, chondrogenic pellets were fixed with 4% PFA and embedded in Tissue-Tek O.C.T.™ Compound. Chondrogenic pellets were sliced into 7 μm thick sections using a freezing microtome (Leica), and then the sections were stained with 0.1% alcian blue.

### Co-culture of ADSCs and bone marrow-derived macrophages

To induce bone-marrow-derived cells into macrophages, 8–10-week-old C57BL/6 mice were euthanized with Isoflurane (5%) + CO_2_ (25%) with a total flow rate of 3 L/min (into a 7 L induction chamber) and then the limbs were removed. The bone marrow cells were flushed from the medullary cavities of femurs and red blood cells were lysed using RBC lysis buffer (Yeasen, China). Next, the bone marrow cells were cultured in RPMI 1640 medium with 10% FBS containing 20 ng/mL recombinant murine M-CSF (Peprotech) and 1% P/S for 7 days to obtain M0 macrophages. For co-culture experiments, M0 type macrophages were digested and re-plated into 6-well plates (5 × 10^5^ cells/well). To induce M1 type macrophage polarization, we stimulated M0 macrophages with 20 ng/mL recombinant murine IFNγ (Peprotech) for 2 days, followed by treatment with 50 ng/mL LPS (Qiagen) for 4 h. To induce type 1 macrophage polarization, we treated M0 type macrophages with 50 ng/mL recombinant murine IL-4 and IL-13 for 48 h. For transwell experiments, 5 × 10^5^ cells ADSCs were resuspended in RPMI 1640 medium with 10% FBS and seeded into the upper chamber with above activated M1 macrophages or M2 macrophages in the lower chamber for 24 h. Finally, we harvested the macrophages for quantitative real-time PCR (qRT-PCR) analysis.

### Quantitative real-time PCR analysis

The total RNA was extracted from cells using Trizol reagent (ThermoFisher Scientific, USA), and it was reverse transcribed into cDNA using the PrimeScript RT reagent kit (Takara, Japan). Real-time PCR (RT-PCR) was performed using SYBR Green Master Mix (Takara, Japan) on a Roche Light Cycler 480 (Roche, Germany). All quantitative experiments were performed at least thrice. The relative amount of each gene was measured with 2^−ΔΔ^CT method. It was normalized by the expression of β-actin. The information of the primer sequences is listed in Additional file 3: Table S1.

### Mitochondrial and lysosomal staining

Mitochondria and lysosomes were stained with Mitotracker Red Dye and LysoTracker Red Dye (ThermoFisher Scientific), respectively, according to the manufacturer's manual. To quantify mitochondrial mass, cells were stained with 50 nM Mitotracker Green (ThermoFisher Scientific) and then were analyzed by flow cytometry.

### Measurement of mitochondrial membrane fluidity

Mitochondria were first isolated using mitochondria isolation kit (Beyotime, Shanghai, China). Mitochondria membrane fluidity was measured by monitoring the fluorescence polarization (*P*) of trimethylammonium 1,6-dipheny-1,3,5-hexatriene dye (TMA-DPH, Medchem Express). The mitochondria were treated with 5 μM TMA-DPH. Fluorescence polarization value was measured using FluoroMax®-Spex 3 spectrofluorometer (Jovin Yvon Horiba, Edison, NJ, USA), at an Ex/Em wavelength of 355/430. *P* was calculated from the fluorescence intensity (FI) according to the following formula:

$$P = \left( {Ivv - G*Ivh} \right)/\left( {Ivv + 2G*Ivh} \right)$$

*Ivv* and *Ivh* represented the excitation and emission fluorescence intensity polarization in vertical (*v*) and horizontal (*h*), respectively. *G* is the apparatus constant dependent on the emission wavelength. *P* value and membrane fluidity are negative correlation.

### Mitochondrial membrane potential assay

Mitochondrial membrane potential of cells was tested with a fluorescent, cell-permeant cationic dye, Tetramethyl rhodamine methyl ester (TMRM), a fluorescent, cell-permeant cationic dye (Beyotime, China) according to the manufacturer’s instruction and then was analyzed by flow cytometry.

### Reactive oxygen species (ROS) assay

ROS levels of cells were evaluated using a fluorescent probe 2′,7′-dichlorofluorescein diacetate (H2-DCFDA) (ThermoFisher Scientific) and then analyzed by flow cytometry.

### Cellular metabolites analysis

When the ADSCs reached 90% confluency, they were washed three times with PBS and then lysed with Metabolite Extraction Buffer (50% Methanol, 30% Acetonitrile, 20% water, with 100 ng/mL HEPES). After that, the cells were collected with a cell scraper, oscillated at 4 °C at 800 rpm for 15 min, pelleted by centrifugation at 4 °C at 13,000*g* for 5 min, and then snap-frozen at − 80 °C. Sextuplicate samples were collected for the LC–MS/MS analysis. The samples were separated by LC ULTRA performance liquid chromatography system (ThermoFisher Ultimate 3000). Then, mass spectrometer (MS, Thermal TSQ QUANTUM μLTRA) was used to analyze the chromatographic peak area and retention time in anion mode. The energy metabolite standard was used to correct the retention time and identify metabolites.

### Seahorse analysis of extracellular acidification rate and oxygen consumption rate

The extracellular acidification rate (ECAR) and Oxygen consumption rate (OCR) were measured using a Seahorse XF96 Extracellular Flux Analyzer (Agilent Technology, Lexington, MA, USA). ADSCs were seeded in 96-well Seahorse plate (1 × 10^4^ per well) and then incubated in DMEM/F12 medium with 5% UltroGro overnight at 37 °C for adhesion. Subsequently, the cells were incubated in basal media containing 1 mM pyruvate, 2 mM glutamine and 10 mM glucose at 37 °C without CO_2_ for 1 h. For the ECAR assay, the ECAR was measured under basal conditions, and after the addition of the following drugs: 5 mM glucose, 1 mM oligomycin and 50 mM 2-DG as indicated. For the OCR assay, the initial OCR was evaluated in basal media as basal respiration, the ATP-linking respiration was measured following the injection of the complex V inhibitor 1 mM oligomycin. The maximal respiration was measured following the injection of 1.5 mM of the uncoupling agent FCCP, then 100 nM rotenone and 1 mM antimycin were added to stop ATP-linking respiration. The cells were repeated four times per group.

### Western blot analysis

ADSCs were lysed using RIPA lysis buffer, and the total protein concentration was measured with a BCA protein assay kit (all from ThermoFisher Scientific). The transferred protein on the PVDF membrane (Millipore) was blocked with 5% non-fat milk for 1 h, followed by incubation with primary antibodies overnight at 4 °C. After washing with TBST, the PVDF membrane was incubated with the corresponding HRP-conjugated second antibody (1:1000, Cell Signaling Technology) at room temperature for 1 h. The primary antibodies used were: β-Actin (1:1,000), Tom20 (1:1000), Hexokinase 1 (HK1, 1:1000); Hexokinase 2 (HK2, 1:1000); Phosphofructokinase (PFK, 1:1000); Lactate dehydrogenase A (LDHA, 1:1000); Citrate Synthase (CS, 1:1000); Dihydrolipoamide succinyltransferase (DLST, 1:1000), Succinate dehydrogenase A (SDHA, 1:1000), and Fumarase (FH, 1:1000) (all obtained from Abcam). Mitochondrial fission factor (MFF, 1:1000); Mitofusin 2 (MFN2, 1:1000); Opticatrophy protein 1 (OPA1, 1:1000) (all obtained from Proteintech).

### Mitochondrial DNA copy number analysis

ADSCs were harvested and total DNA was extracted using a TIANamp Genomic DNA Kit (TIANGEN, China) following the manufacturer’s instructions. The mitochondrial DNA (mtDNA) copy number of cells was analyzed by quantitive RT-PCR, as described previously [32], with nuclear gene (nDNA) used as an internal control. All samples were repeated at least three times.

### Analysis of lysosomal endocytosis

To measure lysosomal endocytosis, ADSCs were cultured in basic medium with FITC-conjugated amyloid peptide (1–42) (FITC-Aβ, PLLABS) for 4 h. After that, the cells were washed with PBS and the cell Nuclei were stained with Hoechst 33,248 (Sigma). The images were then visualized using an inverted fluorescence microscope, and ImageJ software was employed for quantification analysis of images.

### Immunofluorescence staining

The cells were fixed with 4% PFA for 10 min, washed with PBS, and then blocked with 10% horse serum for 30 min. Subsequently, cells were stained with primary antibodies against LAMP-1 and LC3 (1:100, Abcam) at 4 °C overnight. Thereafter, cells were washed with PBS and incubated with second antibodies donkey anti-rabbit conjugated with Alexa Fluor 488 (1:200) and donkey anti-mouse conjugated with Alexa Fluor 594 (1:400, all from Thermo Fisher, Scientific) for 90 min at room temperature. DAPI (Sigma) was used to stain the cell nuclei. The images were observed using an inverted fluorescence microscope, and ImageJ was used for image quantification.

### RNA sequencing

ADSCs were harvested, and total RNA was extracted using TRIzol solution (TIANGEN, China). The concentration of RNA was measured using Nanodrop 2000 and evaluated using Agilent 2100 (LabChip GX). mRNA was purified using mRNA Capture Beads (Vazyme, China), reverse transcribed into cDNA using the Script RT reagent kit (Vazyme, China), and then synthesized into dsDNA. The dsDNA library was constructed using VAHTS Universal V6 RNA-seq Library Prep Kit (Vazyme) and purified using VIHTS DNA Clean Beads (Vazyme) according to the manufacturer’s instructions. The qualified dsDNA samples were sequenced using Illumina Nova Seq 6000 platform (San Diego, USA). Raw sequencing reads were mapped to GRCh38 assembly of the human genome using Tophat2, version 2.0.10 [33]. Fragments per kilobase of exon model per million mapped reads (FPKM) were calculated using Stringtie and normalized with TMM [34–36]. Differentially expressed genes (DEGs) were analyzed using the DESeq 2 package, version 1.10.1 [37]. Genes with log-fold change > 1.5 and false discovery rate (FDR) < 0.05 were considered significant transcriptomic changes. This sequencing dataset is available at GEO: GSE222263.

### Statistical analysis

All data are presented as the mean ± SEM derived from at least three independent experiments. Statistical significance was assessed using Student T-test or one-way ANOVA. *P* ≤ 0.05 was considered statistically significant.

## Results

### Characterization of O-ADSCs and their proliferation ability

**Fig. 1:**
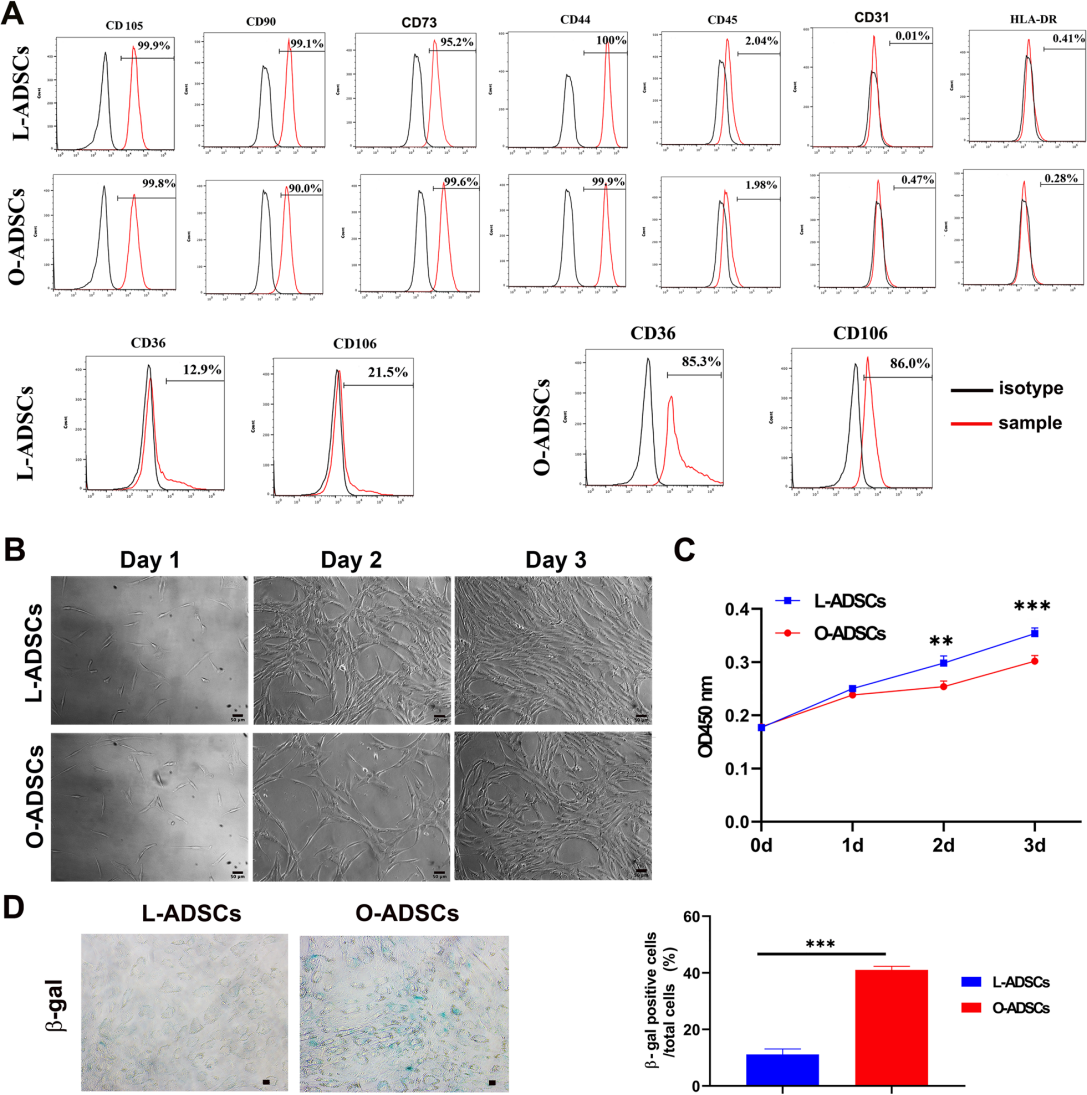
Cell surface marker expression and proliferation potential of L-ADSCs and O-ADSCs. **a** Cell surface marker expression of L-ADSCs and O-ADSCs. **b** Phase contrast images of L-ADSCs and O-ADSCs at day 1, day 2 and day 3. Scale bar, 50 μm. **c** Cell proliferation potential was measured by CCK8 kit (*n* ≥ 3). **d** Represent images of β-gal staining of L-ADSCs and O-ADSCs (left panel) and quantification of β-gal positive cells in total L-ADSCs or O-ADSCs. At least six representative visual fields for each were counted. Scale bar, 50 μm. **P < 0.01; ***P < 0.001 compared to L-ADSCs

First, we isolated L-ADSCs and O-ADSCs derived from the subcutaneous fat tissues of lean donors and obese donors, respectively, following a previously described protocol [38]. Subsequently, L-ADSCs and O-ADSCs were cultured until passage 3 (P3) or passage 4 (P4). Flow cytometry analysis of cell surface markers showed that L-ADSCs and O-ADSCs expressed similar expression levels of cell markers associated with stromal cells (CD105, CD90, CD73 and CD44), and expressed very few cell markers of hematopoietic cells (CD31 and CD45) and HLA-ABC (Fig. 1a). However, O-ADSCs expressed higher levels of CD36 and CD106 (85.3% and 86%, respectively) compared to L-ADSCs, which exhibited lower expression of CD36 and CD106 (12.9% and 21.5%, respectively) (Fig. 1a). We then evaluated the morphology and proliferation capacity of O-ADSCs and L-ADSCs at P3, both O-ADSCs and L-ADSCs exhibited a spindle-shaped morphology (Fig. 1b). However, O-ADSCs showed a slower proliferation capacity than L-ADSCs (Fig. 1b, c). Furthermore, O-ADSCs displayed more β-gal staining, a senescent phenotype, than L-ADSCs (Fig. 1d). In conclusion, these findings suggest that O-ADSCs display altered immunophenotypic profiles, low proliferation ability, and a senescent phenomenon.

### Reduced differentiation potential in O-ADSCs

**Fig. 2:**
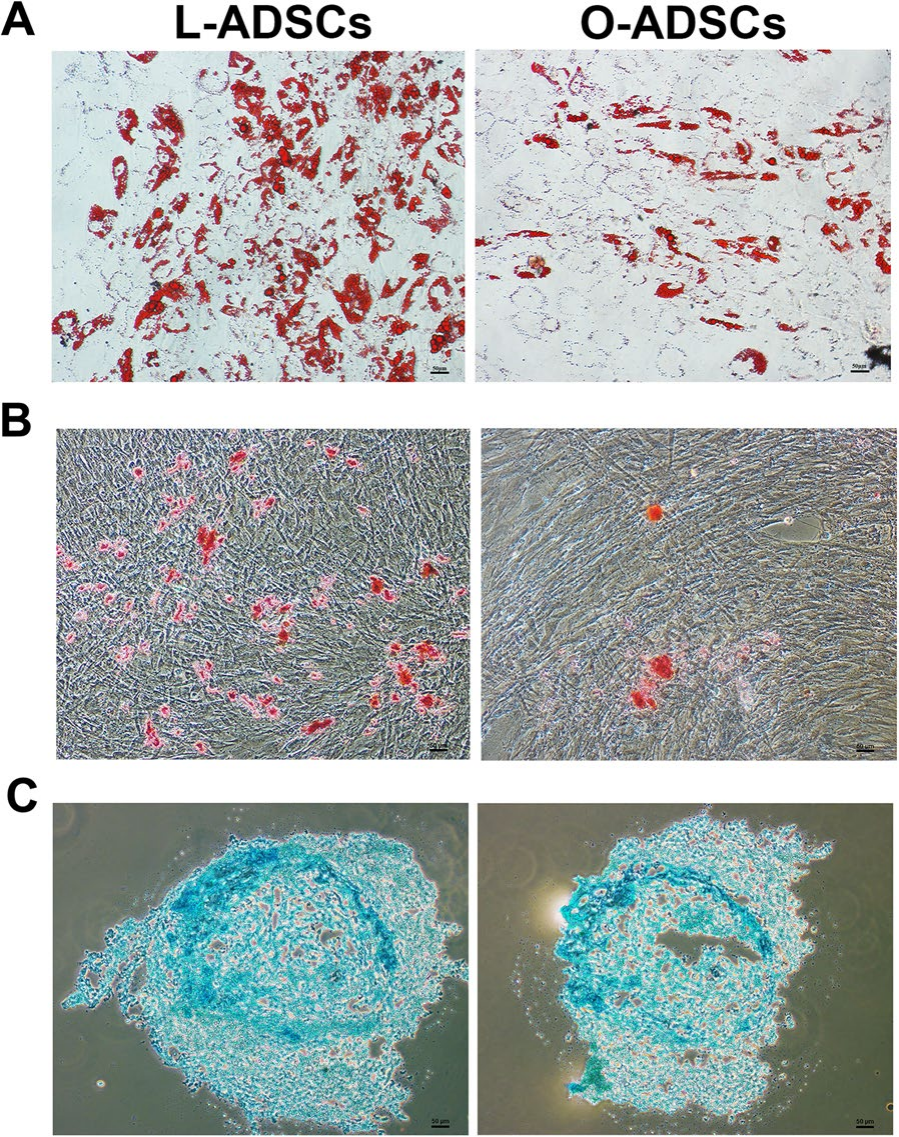
In vitro differentiation potential of L-ADSCs and O-ADSCs. **a** ADSCs were differentiated into adipocytes and then analyzed by Oil red O staining. Scale bar, 50 μm. **b** Alizarin red staining was used to evaluate osteogenesis differentiation potential of L-ADSCs and O-ADSCs. Scale bar, 50 μm. **c** ADSCs were differentiated into chondrocytes and then analyzed by Alcian blue. Scale bar, 50 μm

To assess the differentiation potential of O-ADSCs and L-ADSCs, we cultured the cells in adipogenic, osteogenic, and chondrogenic differentiation mediums, respectively. Then, the adipogenic, osteogenic and chondrogenic potential of ADSCs were measured by oil red staining, alizarin red and alcian blue, respectively. The results showed that O-ADSCs had fewer adipose droplets and bone-like nodules compared to L-ADSCs (Fig. 2a, b). Furthermore, O-ADSCs produced fewer chondrocytes than L-ADSCs when the cell numbers were similar before induction (Fig. 2c). These findings suggest that O-ADSCs have weaker differentiation potential than L-ADSCs.

### O-ADSCs promote M1-type macrophage polarization and increase the pro-inflammatory phenotype

**Fig. 3:**
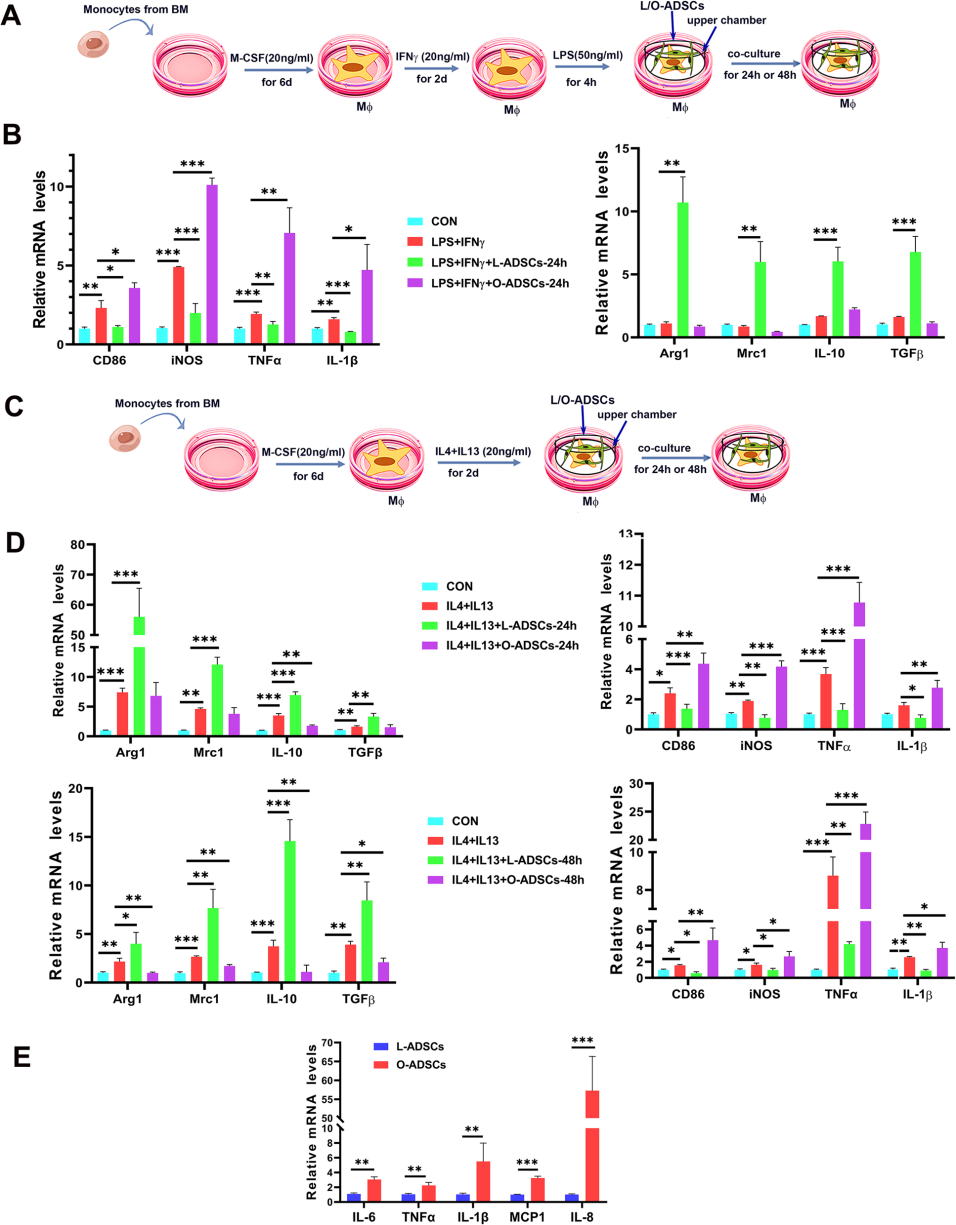
Macrophage polarization and inflammatory cytokines secretion ability of L-ADSCs and O-ADSCs. **a** A schematic illustration of experimental design for in vitro M1-like macrophages co-culture with ADSCs. BM represents bone marrow. Mφ represents macrophage.** b** The mRNA levels of M1-like macrophage markers and M2-like macrophage markers of macrophages after M1-like macrophages co-cultured with L-ADSCs and O-ADSCs for 24 h. **c** A schematic illustration of experimental design for in vitro M2-like macrophages co-culture with ADSCs. **d** The mRNA levels of M2-like macrophage markers and M1-like macrophage markers of macrophages after M2-like macrophages co-cultured with L-ADSCs and O-ADSCs for 24 h or 48 h. **e** mRNA levels of inflammatory cytokines IL-6, TNFα, IL-1α, MCP1, IL-8 in L-ADSCs and O-ADSCs. Data were collected from at least three independent experiments, and the data are expressed as the mean ± SEM. *P < 0.05; **P < 0.01; ***P < 0.001

Obesity is associated with a low-grade chronic inflammatory state in which M1-type macrophages play an important role [39, 40]. To investigate the effect of O-ADSCs on macrophage differentiation and polarization, we isolated monocytes from mouse bone marrow and induced them to differentiate into naïve (M0 type) macrophages with M-CSF. Next, M0 type macrophages were polarized into pro-inflammatory M1 macrophages with IFNγ plus LPS for 24 h, followed by co-culture with L-ADSCs or O-ADSCs for 24 h (Fig. 3a). The resulting macrophages were harvested for RT-PCR. As shown in Fig. 3b, the mRNA levels of M1-type-related markers including CD86, iNOS, TNFα and IL-1β were decreased in macrophages when co-cultured with L-ADSCs for 24 h, while the expression levels of M2-type-related genes including Arg-1, Mrc1(CD206), IL-10 and TGFβ were increased. In contrast, macrophages co-cultured with O-ADSCs showed a significant increase in the mRNA levels of M1-related markers, while no significant changes were observed in the expression levels of M2-related genes. To further investigate this phenomenon, we induced mouse bone marrow M0 type macrophages to polarize into M2 macrophages with IL-4 and IL13, and then co-cultured the M2 type macrophages with L-ADSCs or O- ADSCs for 24 h or 48 h (Fig. 3c). As shown in Fig. 3d, L-ADSCs remarkably increased the expression of M2-related marker genes including Arg1, Mrc1, IL-10, and TGF β in macrophages (Fig. 3d). Conversely, O-ADSCs failed to activate M2-related gene expression of macrophages, even exhibited an inhibitory effect on the M2-related gene expression in macrophages when co-cultured for 48 h (Fig. 3d). Of note, O-ADSCs significantly enhanced M1-related gene expression, indicating that O-ADSCs promote M1-type macrophage polarization from M2-type macrophages. In summary, these results suggest that L-ADSCs promote macrophage polarization from M1-type to M2-type, while O-ADSCs enhance macrophage polarization from M2-type to M1-type.

To measure the expression of inflammatory cytokines and chemokines in ADSCs, we performed RT-PCR. As shown in Fig. 3e, the expression levels of pro-inflammatory cytokines and chemokines including IL-6, TNFα, IL-1β, MCP-1 and IL-8 were significantly higher in O-ADSCs than those in L-ADSCs. These findings suggest that O-ADSCs promote the production of pro-inflammatory cytokines and chemokines, which in turn contribute to the maintenance of the inflammatory environment of adipose tissues in obesity. Furthermore, these results support our earlier observations that O-ADSCs promote M1 macrophage polarization, indicating their role in perpetuating chronic low-grade inflammation.

### The mitochondrial morphology and function of O-ADSCs are impaired

**Fig. 4:**
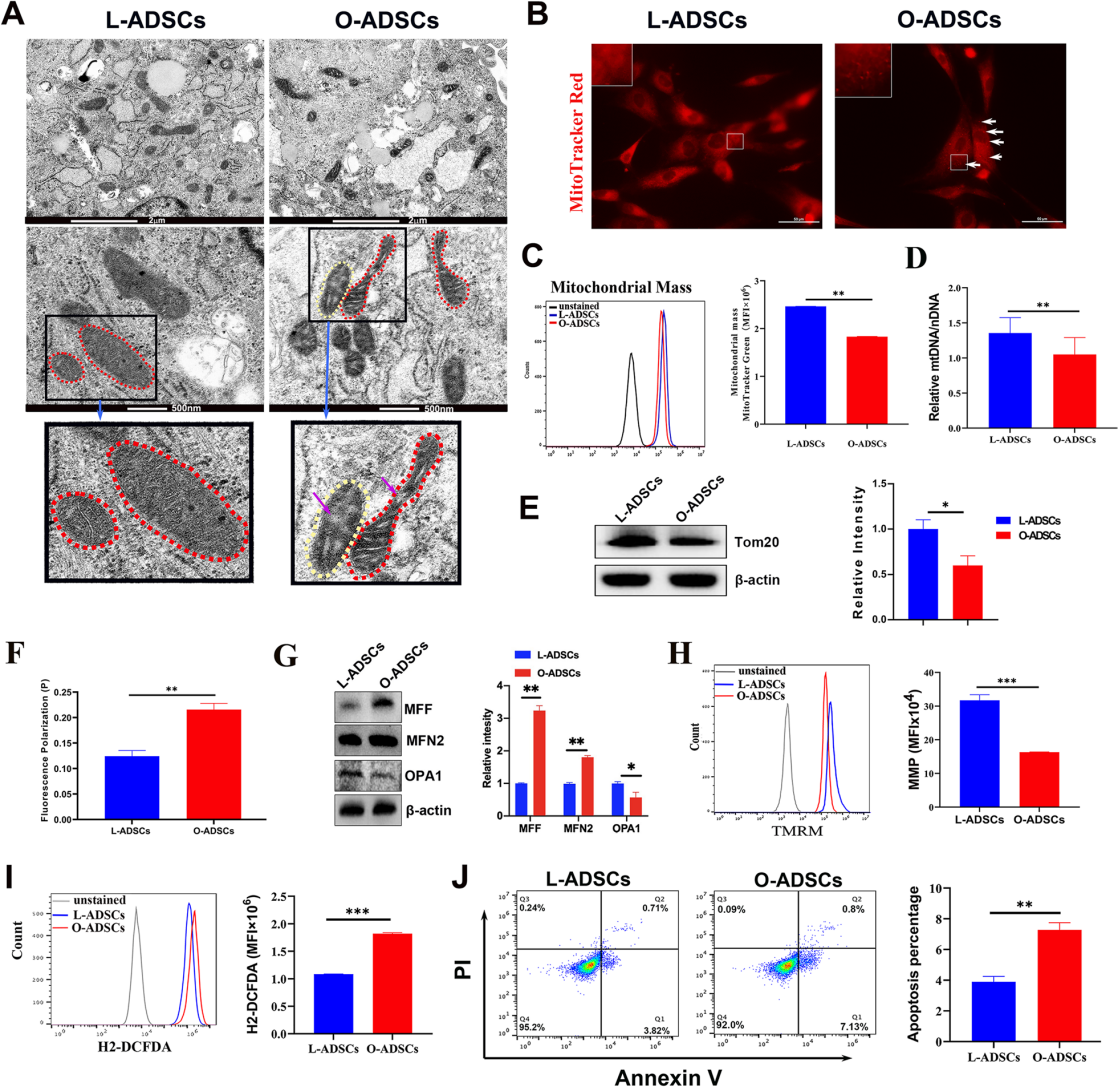
Mitochondrial morphology and function of L-ADSCs and O-ADSCs. **a** Representation of TEM images of the mitochondrial of L-ADSCs and O-ADSCs. The mitochondrial structure of L-ADSCs was well organized (red dotted coil). In contrast, that of O-ADSCs were slim (yellow dotted coil) and disordered (red dotted coil) and some swollen regions were devoid of cristae (purple arrow). Scale bar, 2 μm (up panel) and 500 nM (middle panel). **b** Representative images of the mitochondria in L-ADSCs and O-ADSCs stained by MitoTracker Red. Some small and punctate fragments (white arrows) were observed in the mitochondria of O-ADSCs. Scale bar, 50 μm. **c** Representative flow cytometry images of mitochondria mass stained by MitoTrack Green and its quantification in L-ADSCs and O-ADSCs (*n* ≥ 3). **d** mtDNA levels in nuclear DNA (nDNA) of L-ADSCs and O-ADSCs by RT-PCR assays. **e** Western blot analysis of mitochondrial membrane protein Tom 20 levels in L-ADSCs and O-ADSCs and their qualification of relative protein intensity. Full-length blots are presented in Additional file 2: Figure S2. **f** Quantification of mitochondrial membrane fluidity of L-ADSCs and O-ADSCs by the fluorescence polarization (*P*) values of mitochondrial membrane measured by TMA-DPH dye. *P* value and membrane fluidity are negative correction (*n* ≥ 3). **g** Western blot analysis of fusion and fission protein level in L-ADSCs and O-ADSCs and their qualification of relative protein intensity. Full-length blots are presented in Additional file 2: Figure S3. **h** Representative flow cytometry images of the mitochondrial membrane potential in L-ADSCs and O-ADSCs detected by TMRM probe and their quantification in L-ADSCs and O-ADSCs (*n* ≥ 3). **i** Flow cytometry analysis of ROS levels by H2-DCFDA dye in L-ADSCs and O-ADSCs and their quantification (*n* ≥ 3). **j** Flow cytometry analysis of apoptotic cells using PI/Annexin V staining in L-ADSCs and O-ADSCs and their quantification (*n* ≥ 3)

Mitochondria play a crucial role in ATP production, fatty acid catabolism through β-oxidation, redox homeostasis, and apoptosis initiation and execution [13, 41–43]. In this study, we investigated the morphological changes in the mitochondria of O-ADSCs and L-ADSCs by examining their mitochondrial structure using transmission electron microscope (TEM). Our results showed that some mitochondria of O-ADSCs became slimmer and disordered, with distinct swollen regions without cristae (arrow) observed (Fig. 4a). We then stained the mitochondria with Mitotracker Red dye and found small punctate fragments in the mitochondria of O-ADSCs, while the mitochondria of L-ADSCs were continuous and uniform (Fig. 4b). Additionally, we measured the mitochondrial mass with MitoTracker Green, and flow cytometry analysis revealed that O-ADSCs had lower mitochondrial mass than L-ADSCs (Fig. 4c). The mitochondrial genome (mtDNA) is a circular, double-free DNA. The copy number of mtDNA is a biomarker of mitochondrial function and is associated with changes in oxidative phosphorylation, oxidative stress, and mitochondrial membrane potential [44, 45]. Therefore, we measured the mtDNA copy number using RT-PCR and found that the copy number was reduced in O-ADSCs compared to L-ADSCs (Fig. 4d). At the same time, the protein level of translocase of outer mitochondrial membrane Tom20 was lower in O-ADSCs than that in L-ADSCs (Fig. 4e and Additional file 2: Fig S2).

In addition, we determined mitochondrial homeostasis by measuring mitochondrial membrane fluidity by a fluorescence dye, trimethylammonium 1,6-dipheny-1,3,5-hexatriene dye (TMA-DPH). The fluorescence polarization (*P*) was calculated from the fluorescence intensity (FI) as following formula: *P* = (*Ivv* − G * *Ivh*)/(*Ivv* + 2G * *Ivh*). *P* value is negative correction with mitochondrial membrane fluidity of cells [46]. Figure 4f shows that the *p* value of O-ADSCs is stronger than that of L-ADSCs, indicating that the mitochondrial membrane fluidity of O-ADSCs is weaker than that of ADSCs. Moreover, we further evaluated the expression of mitochondrial fission and fusion proteins by western blot. As shown in Fig. 4g and Additional file 2: Fig S3, the expression levels of mitochondrial fission proteins MEF and MFN2 are higher in O-ADSCs than those of L-ADSCs, while protein levels of mitochondrial fusion protein OPA1 is lower in O-ADSCs, indicating the balance of mitochondrial fusion and fission proteins is broken in O-ADSCs. Next, we measured mitochondrial membrane potential (MMP) by a mitochondrial selective probe tetramethyl rhodamine methyl ester (TMRM) and found that MMP of O-ADSCs was lower than that of L-ADSCs (Fig. 4h). We also assessed the levels of reactive oxygen species (ROS) in O-ADSCs and L-ADSCs using an oxidant-sensing fluorescent probe H2-DCFDA. As shown in Fig. 4i, the level of ROS was higher in O-ADSCs than in L-ADSCs. Additionally, we assessed the apoptosis of ADSCs with PI and Annexin V and found that apoptotic cells in O-ADSCs were increased than those in L-ADSCs (Fig. 4j). In conclusion, these data indicate that the mitochondrial phenotype was altered, and the mitochondrial homeostasis and function was impaired.

### The reduction of energy metabolism in O-ADSCs

**Fig. 5:**
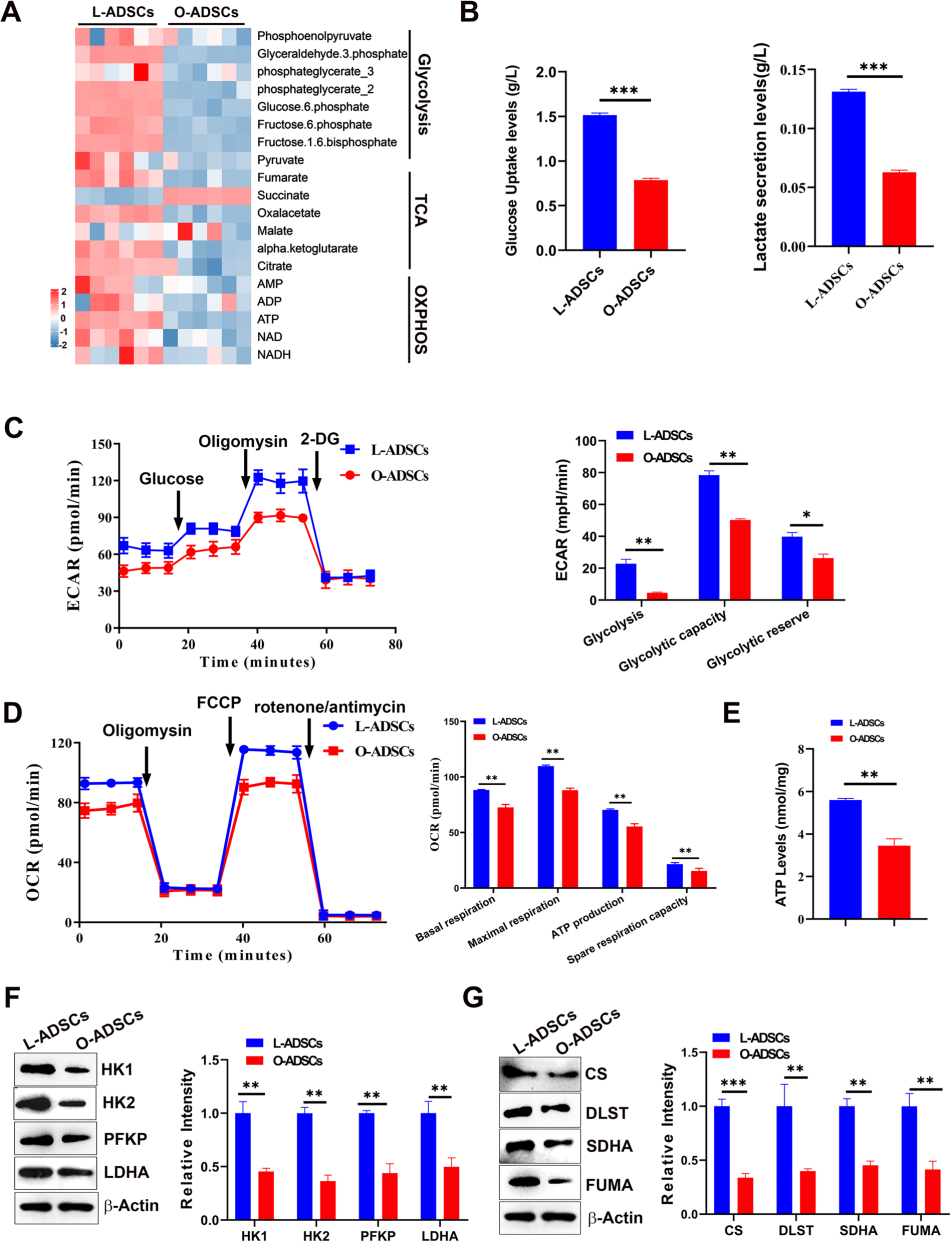
Analysis of metabolites and enzymes in glycolysis and TCA in L-ADSCs and O-ADSCs. **a** Heatmap of cellular metabolites in O-ADSC versus L-ADSCs. **b** The concentration of glucose uptake and lactate secretion levels in the supernatant in L-ADSCs and O-ADSCs after culture for 24 h (*n* ≥ 3). **c** ECAR in L-ADSCs and O-ADSCs and qualification of ECAR processes. **d** OCR in L-ADSCs and O-ADSCs and their qualification in OCR processes. **e** ATP levels in the supernatant in L-ADSCs and O-ADSCs after culture for 24 h (*n* ≥ 3). **f, g** Western blot analysis of the protein levels of glycolysis related enzymes and TCA related enzymes and their qualification of relative protein intensity. Full-length blots are presented in Additional file 2: Figure S4 and S5

Mitochondria are crucial for generating ATP through electron transport and oxidative phosphorylation, which are coupled with the tricarboxylic acid (TCA) cycle [47]. To investigate the changes in energy metabolism in O-ADSCs, we analyzed intracellular energy metabolites of O-ADSCs and L-ADSCs targeting 19 metabolites. The heat map indicated that most metabolites were significantly downregulated in O-ADSCs (*P* < 0.05), including those from glycolysis, and the TCA cycle (Fig. 5a, Additional file 4: Table S2). Moreover, the levels of ATP, ADP, and NADH, which drive OXPHOS to produce ATP, were decreased in O-ADSCs (*P* < 0.05) (Fig. 5a). To confirm the change in glucose metabolism of O-ADSCs, we tested glucose uptake and lactate secretion. As shown in Fig. 5b, the glucose uptake and lactate secretion were decreased in O-ADSCs than in L-ADSCs. These results suggest that energy metabolism, including glycolysis and the TCA cycle, was reduced in O-ADSCs. To confirm this, we measured the extracellular acid rate (ECAR) of ADSCs using Seahorse metabolic analyzer to assess glycolysis. Figure 5c shows that O-ADSCs exhibited a lower ECAR than L-ADSCs. Next, we tested the oxygen consumption rate (OCR) to determine OXPHOS of ADSCs. As shown in Fig. 5d, the OCR was lower in O-ADSCs than in L-ADSCs. Comparable results were observed for ATP production in the supernatant in O-ADSCs. We also measured the levels of enzymes involved in the glycolytic pathway (HK1, HK2, PFKP, and LDHA) and TCA cycle (CS, DLST, SDHA, and FUMA) by western blot and found their expression levels were lower in O-ADSCs than those in L-ADSCs (Fig. 5f, g, Additional file 2: Fig S4 and S5, respectively), indicating that the activity of the glycolytic pathway and TCA cycle in O-ADSCs was downregulated. Collectively, the data indicated that the energy metabolism of mitochondria is decreased in O-ADSCs.

### Lysosome function of O-ADSCs was impaired

**Fig. 6:**
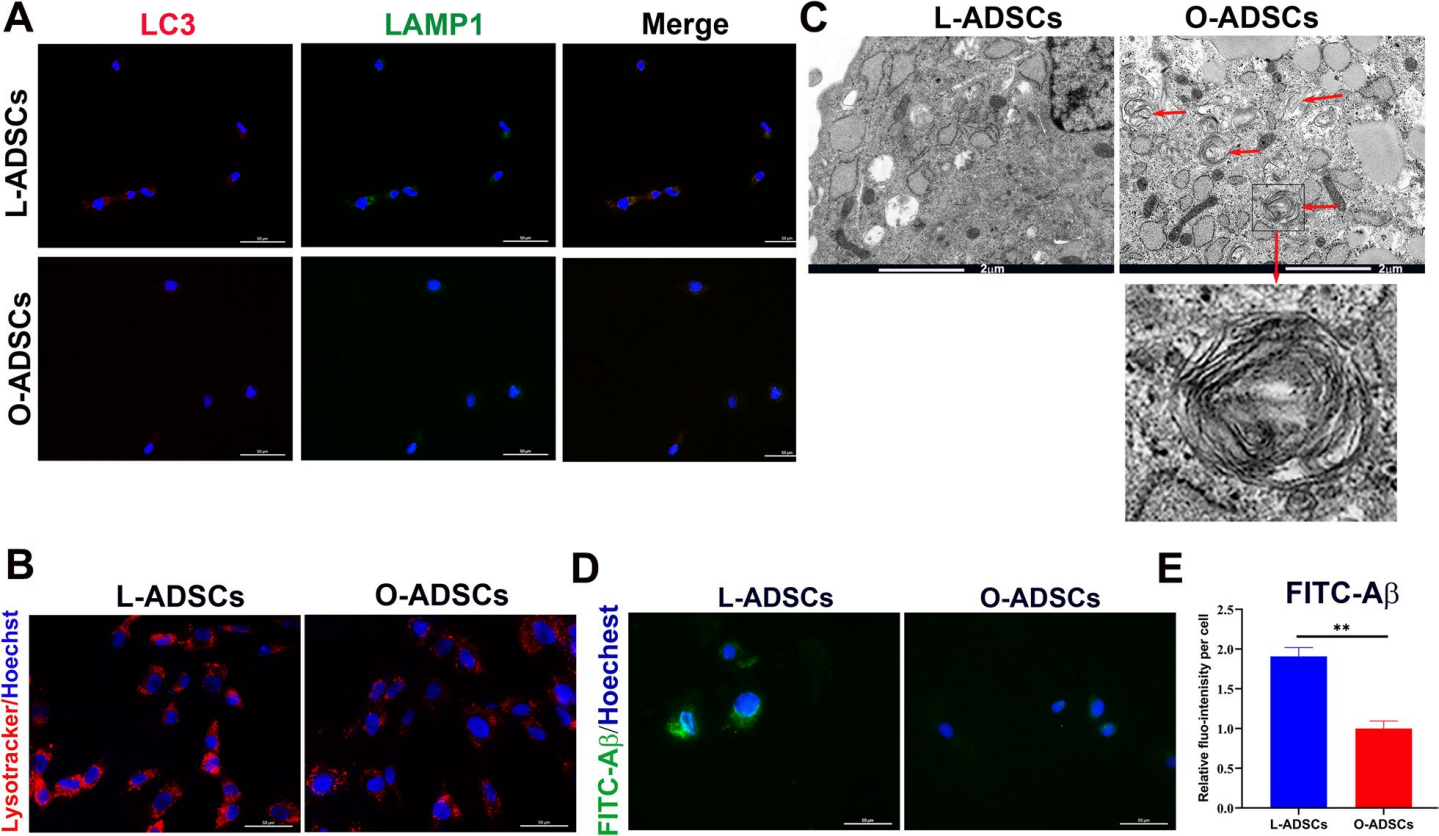
Lysosomal morphology and function were impaired in O-ADSCs. **a** Immunofluorescence staining of L-ADSCs and O-ADSCs with antibodies against LC3 and LAMP1. Scale bar, 50 μm. **b** Representative lysosomal images of L-ADSCs and O-ADSCs stained with LysoTracker Red. Scale bar, 50 μm. **c** Representative TEM images of L-ADSCs and O-ADSCs. Red arrows indicate atypical lysosomes with some undegraded materials. Scale bar, 2 μm. **b** Immunofluorescence staining of L-ADSCs and O-ADSCs with antibodies against LC3 and LAMP1. Scale bar, 50 μm. **c** Representative lysosomal images of L-ADSCs and O-ADSCs stained with LysoTracker Red. Scale bar, 50 μm. **d** Phagocytosis of L-ADSC and O-ADSCs was measured by phagocytic FITC-Aβ after being treated with FITC-Aβ for 4 h. **e** Qualification of fluorescence(fluo-) intensity of FITC-Aβ per cell in L-ADSCs and O-ADSCs. At least 50 cells were assessed in each group

Autophagy plays an essential role in maintaining cellular and organismal homeostasis by protecting against various stress insults; however, obesity can often interfere with the autophagic process [48]. To evaluate the autophagy ability of O-ADSCs, we performed immunofluorescence with LC3, a marker of autophagy, and lysosome-associated membrane glycoprotein 1 (LAMP1). As shown in Fig. 6a and Additional file 1: Fig. S1a shows that LC3 and LAMP1 displayed strong fluorescence signals in L-ADSCs, while they showed weak fluorescence signals in O-ADSCs. This result indicated that O-ADSCs had a decreased autophagy ability. Moreover, we also used Lysotracker red to label lysosomes and found that while L-ADSCs exhibited a strong perinuclear staining, O-ADSCs showed a weaker diffusing staining, implying a change in the lysosomal membrane permeabilization of O-ADSCs (Fig. 6b). Autophagy is a major function of lysosomes [49], and therefore, we further investigated the lysosomal function in O-ADSCs. We first performed TME to observe the morphological change of lysosomes in ADSCs. As shown in Fig. 6c, there were some lysosomes containing undegraded materials in O-ADSCs (red box), indicating a decline in the degradation ability of lysosomes in O-ADSCs. To evaluate the lysosomal phagocytic ability of ADSCs, we cultured ADSCs with FITC-amyloid-beta (FITC-Aβ). As shown in Fig. 6d, e and Additional file 1: Fig. S1b, L-ADSCs accumulated more FITC-Aβ deposition than O-ADSCs, suggesting that the phagocytic capacity of lysosomes in O-ADSCs was decreased. Overall, the above findings revealed that the lysosomal function in O-ADSCs was impaired.

### Gene expression profile of O-ADSCs and L-ADSCs

**Fig. 7:**
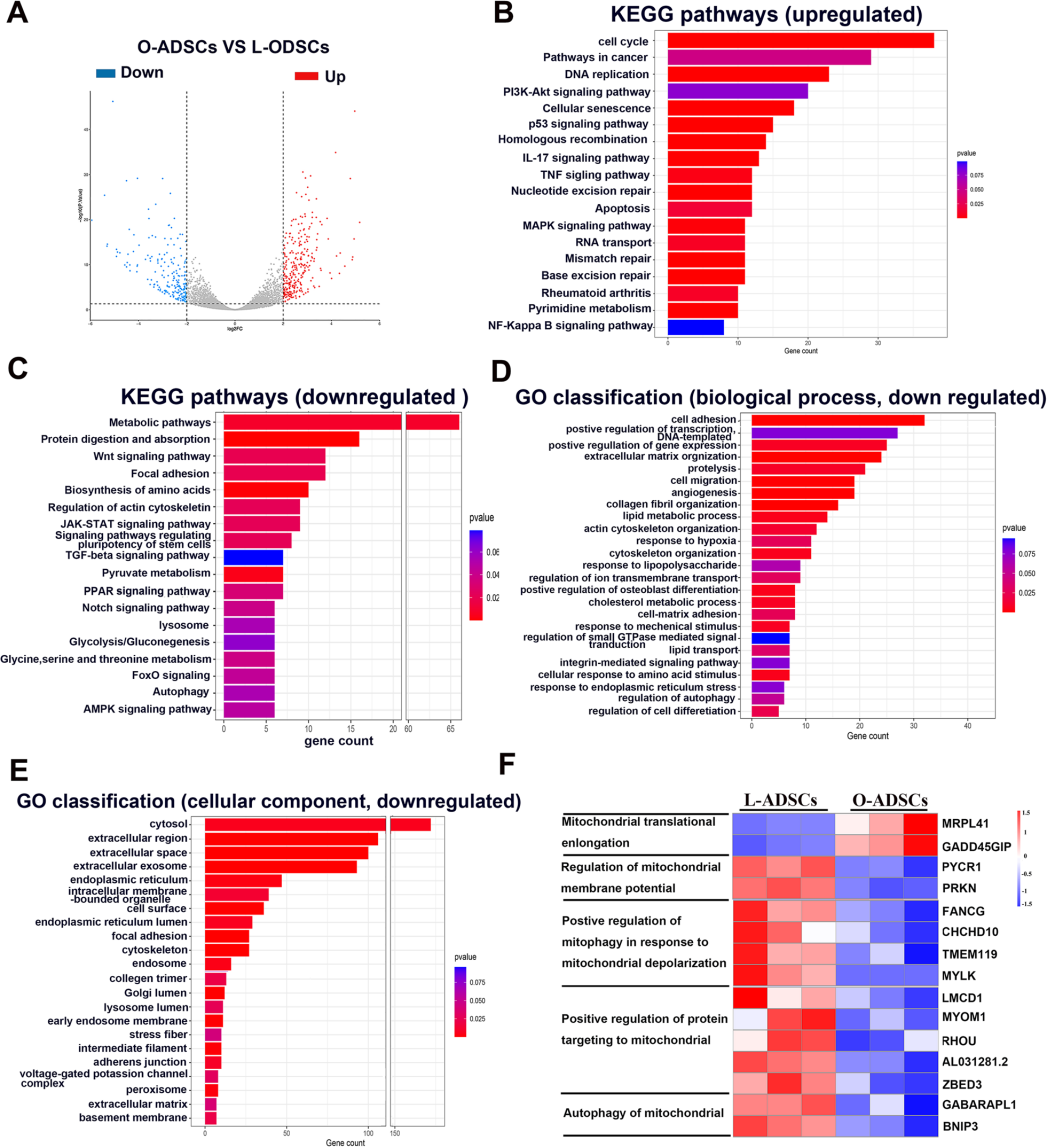
RNA sequencing analysis of L-ADSCs and O-ADSCs. a volcano plot of gene expression upregulated and downregulated in O-ADSCs versus L-ADSCs. **b**,** c** KEGG pathway enrichment analysis of the upregulated and downregulated pathways in O-ADSCs versus L-ADSCs. **d**, **e** Gene Ontology (GO) classification analysis of downregulated genes changes in biological process and cellular components in O-ADSCs versus L-ADSCs. **f** Heat map of mitochondria-related gene expression upregulated and downregulated in O-ADSCs versus L-ADSCs

To determine changes in the gene expression profiles of O-ADSCs, we compared the transcript profiles of O-ADSCs and L-ADSCs using RNA sequencing. Based on gene difference (fold change ≥ 1.5) and statistical significance analysis (*P* value ≤ 0.05), the volcano plot displayed the differentially expressed genes (Fig. 7a). Next, the differentially expressed genes were subjected to KEGG pathway enrichment analysis and Gene Ontology (GO) classification. As shown in Fig. 7b, the pathways related to cancer, cellular senescence, apoptosis, and inflammatory responses in O-ADSCs were upregulated. However, the pathways related to metabolism, glycolysis, biosynthesis and metabolism of amino acid, AMPK signaling, stemness of stem cells, lysosome, autophagy, and focal adhesion in O-ADSCs were downregulated (Fig. 7c). These results are consistent with our previous findings (Figs. 3, 4, 5, 6) that metabolism, energy metabolism, anabolism, stemness, and lysosome were downregulated, while cellular senescence, apoptosis, and inflammation were upregulated.

The GO classification analysis showed that the biological processes associated with cell adhesion, cell migration, cell differentiation, lipid metabolism and transport process, and response to external stress and stimulus were less sensitive in O-ADSCs compared to those in L-ADSCs (Fig. 7d). Further GO analysis confirmed that the cellular components associated with cytosol, organelle including endosome, lysosome, exosome, Golgi, endoplasmic reticulum, cellular skeleton, adhesion, and extracellular matrix in O-ADSCs were reduced (Fig. 7e). As for mitochondria, the genes related to translation elongation were upregulated in O-ADSCs, however, the regulation of mitochondrial membrane, mitophagy, and mitochondrial protein were reduced in O-ADSCs (Fig. 7f). This may lead to an imbalance of mitochondrial fission and fusion, disrupting mitochondrial dynamics and homeostasis. These results suggest that mitochondrial and lysosomal functions are impaired in O-ADSCs, which affect cellular responses to external stimulus and stress [16, 50], as well as cellular biological function.

## Discussion

In this study, our findings revealed that O-ADSCs exhibited higher expression levels of CD36 and CD106 (Fig. 1a). CD36, a transmembrane glycoprotein, plays a key role in the uptake of long-chain fatty acids [51], while CD106 is involved in immune responses and the recruitment of leukocytes to inflammatory sites by interacting with integrin alpha-4/beta-1 [52]. Therefore, the increased expression of CD36 and CD106 in O-ADSCs could contribute to fatty acid uptake and inflammation in obese adipose tissue. In addition, our results have shown that L-ADSCs inhibit pro-inflammatory M1 type macrophage polarization and promote anti-inflammatory M2 type macrophage polarization. On the contrary, O-ADSCs promote pro-inflammatory M1 type macrophage polarization (Fig. 3). Therefore, in obese adipose tissue, O-ADSCs may play a crucial role in activating and maintaining M1-type macrophages, which could contribute to the development and maintenance of obesity and obesity-related diseases.

Intriguingly, we found that the mitochondrial structure of O-ADSCs was altered, as indicated by the morphological changes of mitochondria with slim size and swollen areas, a reduction in mitochondrial mass and mtDNA. We also observed the mitochondrial homeostasis or dynamics was disrupted, as indicated by decreased mitochondrial membrane fluidity, the broken balance of fusion/fission, increased mitochondrial fragmentation in O-ADSCs. Previous study showed that excess mitochondrial fission may promote mitochondrial dysfunctions and lead to cell injury [53, 54]. The data reveal that impaired mitochondrial morphology and structure lead to mitochondrial dysfunction, which leads to cell injury in O-ADSCs and ultimately to the reduction of cell activity.

As for the mechanism of the mitochondrial dysfunction and reduced activity of O-ADSCs, we found that glucose uptake, glycolysis, ECAR, OCR, glycolysis- and TCA-related enzymes, and their intermediate metabolites are decreased in O-ADSCs compared to those in L-ADSCs. The data indicated the mitochondrial function are impaired in O-ADSCs. Of note, the TCA cycle is the common oxidative pathway for carbohydrates, fatty acids, and amino acid, and generate energy compound ATP and GTP, and intermediate metabolites needed for many biosynthetic process of cell, such as protein synthesis, de novo DNA and fatty acid synthesis [12, 55]. Eventually, mitochondrial dysfunction impairs cell structure and function, leading to the reduction of biological activity of O-ADSCs. On the other hand, impaired mitochondria increase ROS production. Previous study reported that mitochondrial ROS promote mitochondrial dysfunction and inflammation. When treating injured cell with Mito-Tempo (MT, a mtROS scavenger), the damaged cells can restore mitochondrial function and inhibit inflammation [56]. Oxidative stress can activate NF-kb signaling pathway, which mediates inflammatory response [57]. Reversely, inflammation also impair mitochondrial biogenesis and function [58]. Moreover, excess ROS impairs lipogenesis of membrane, leading to the disruption of mitochondrial membrane. As for the mitochondrial regulatory pathway, RNA sequencing of O-ADSCs and L-ADSCs showed that AMPK pathway, which can activate PGC-1α [59], was downregulated, while reduced PGC-1α decreases mitochondrial biogenesis and mitochondrial function. Therefore, in obesity, excess ROS production, inflammation, and downregulated AMPK pathway promote mitochondrial dysfunction of O-ADSCs, resulting in the reduced biological function of O-ADSCs.

In addition to mitochondrial dysfunction, we also evaluated the lysosomal morphology and function of O-ADSCs. We found that lysosomal membrane permeabilization in O-ADSCs was increased, some lysosomes of O-ADSCs contained accumulated undegraded materials, which is a pathogenic phenomenon in some lysosome storage diseases and neurodegenerative diseases [60, 61]. Additionally, the autophagy ability and phagocytosis of O-ADSCs were decreased. Previous studies demonstrated that mitophagy regulate mitochondrial network, oxidative stress, and apoptosis [62]. These studies reveal that decreased autophagy ability impairs mitochondrial function and cell activity. Additionally, cell senescence is characterized by a gradual structural change of lysosomes, accompanied by a decrease in lysosome function, which is termed as age-related lysosome damage [63]. Therefore, we believe that the lysosome dysfunctions in O-ADSCs cause the accumulation of cellular waste and damaged organelles, affecting cellular components recycling and disturbing cell homeostasis, which eventually leads to a decline in cellular biological activity [59].

## Conclusions

Our study revealed that O-ADSCs exhibit a decline in their cellular biological activity compared to L-ADSCs. Importantly, we found that O-ADSCs exhibit mitochondrial and lysosomal dysfunctions, may leading to the reduction of biological activity of O-ADSCs. Therefore, targeting the mitochondrial and lysosomal dysfunction of O-ADSCs can be an effective therapeutic strategy to improve their cell activity, which is also be beneficial to the treatment of obesity and its related disorders.

### Supplementary Information

**Additional file 1**：Figure S1. Lysosomal morphology of L-ADSCs and O-ADSCs from more donors and the representative images are shown. a Immunofluorescence staining of L-ADSCs and O-ADSCs with antibodies against LC3 and LAMP1. Scale bar, 50 μm. b Phagocytosis of L-ADSC and O-ADSCs was measured by phagocytic FITC-Aβ after being treated with FITC-Aβ for 4 hours. Scale bar, 50 μm.

**Additional file 2**：Figure S2–S5. Uncropped full-length blots of western blot images.

**Additional file 3**. Table S1. Primers used for real-time PCR.

**Additional file 4**. Table S2. Relative significant expression level changes of intermediate metabolites related energy metabolism in the cell lysate of L-ADSCs and O-ADSCs. Related to Figure 5A.

## Data Availability

RNA sequencing datasets have been deposited in the GEO: GSE222263 and is publicly available. All related data and materials are available under request.

## Abbreviations

ADSCs: Adipose-derived stem cells
TEM: Transmission electron microscope
TMRM: Tetramethyl rhodamine methyl ester
ROS: Reactive oxygen species
HK1: Hexokinase 1
HK2: Hexokinase 2
PFK: Phosphofructokinase
LDHA: Lactate dehydrogenase A
CS: Citrate synthase
DLST: Dihydrolipoamide succinyltransferase
FH: Fumarase
SDHA: Succinate dehydrogenase A
TCA: Tricarboxylic acid
OXPHOS: Oxidative phosphorylation
LC–MS/MS: Liquid chromatography–mass spectrometry/mass spectrometry
LAMP1: Membrane glycoprotein 1
Aβ‐FITC: FITC‐beta amyloid
GO: Gene ontology
