# Supplementary figures and images for: Uncovering impaired mitochondrial and lysosomal function in adipose-derived stem cells from obese individuals with altered biological activity
Source: Stem Cell Res Ther. 2024 Jan 8;15:12. doi: 10.1186/s13287-023-03625-9 (PMC10773039; doi:10.1186/s13287-023-03625-9)

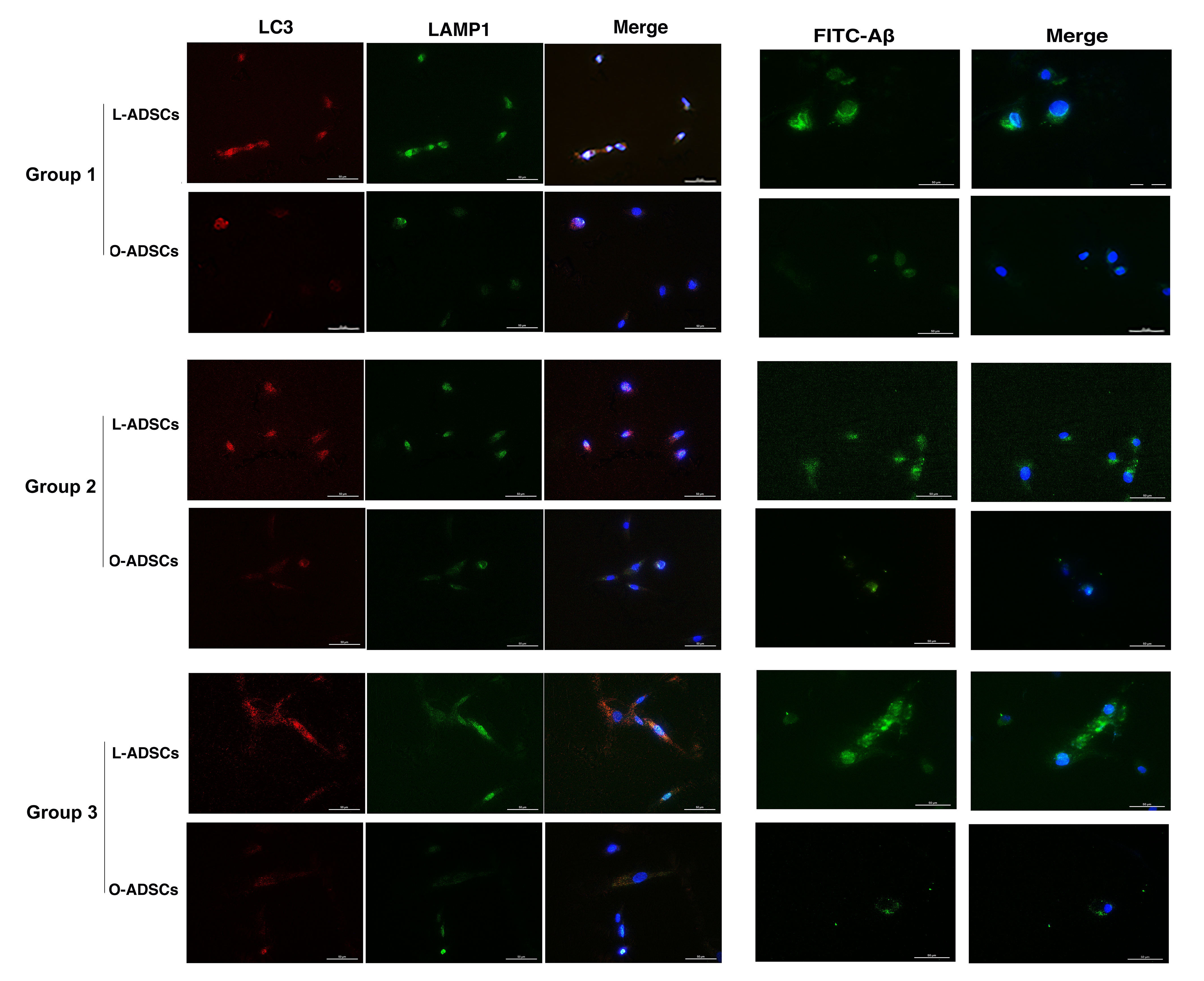

Supplement: Supplementary file 1 — Additional file 1：Figure S1. Lysosomal morphology of L-ADSCs and O-ADSCs from more donors and the representative images are shown. a Immunofluorescence staining of L-ADSCs and O-ADSCs with antibodies against LC3 and LAMP1. Scale bar, 50 μm. b Phagocytosis of L-ADSC and O-ADSCs was measured by phagocytic FITC-Aβ after being treated with FITC-Aβ for 4 hours. Scale bar, 50 μm. [file 13287_2023_3625_MOESM1_ESM.tif]
