# Supplementary material for: Uncovering impaired mitochondrial and lysosomal function in adipose-derived stem cells from obese individuals with altered biological activity
Source: Stem Cell Res Ther. 2024 Jan 8;15:12. doi: 10.1186/s13287-023-03625-9 (PMC10773039; doi:10.1186/s13287-023-03625-9)
Supplement: Supplementary file 2 — Additional file 2：Figure S2–S5. Uncropped full-length blots of western blot images. [file 13287_2023_3625_MOESM2_ESM.docx]

**Uncropped full-length blots**

**Figure S2. Full-length blots of western blot images of figure 4E.**


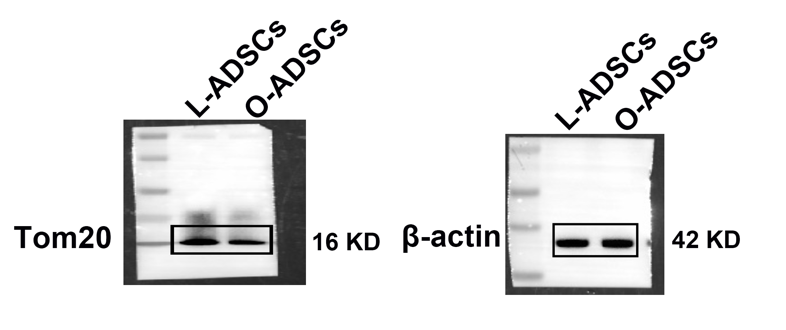


**Figure S3. Full-length blots of western blot images of figure 4G.**


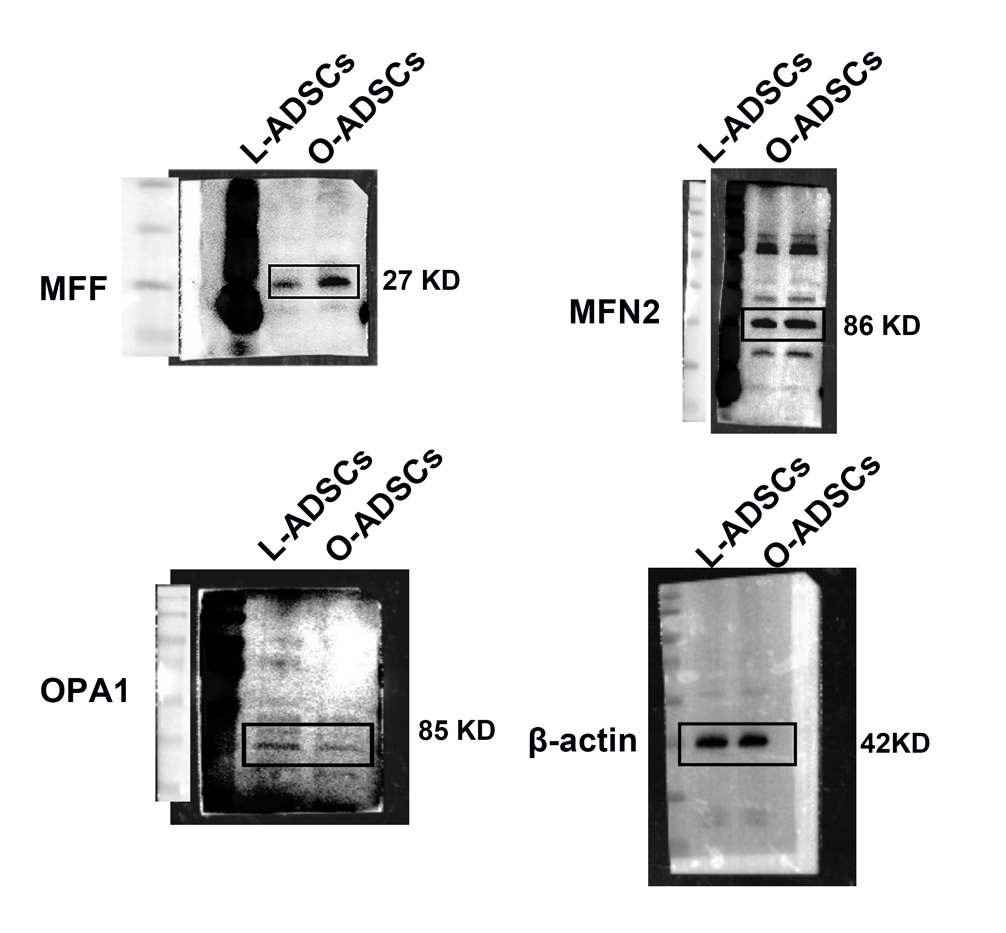


**Figure S4. Full-length blots of western blot images of figure 5F.**


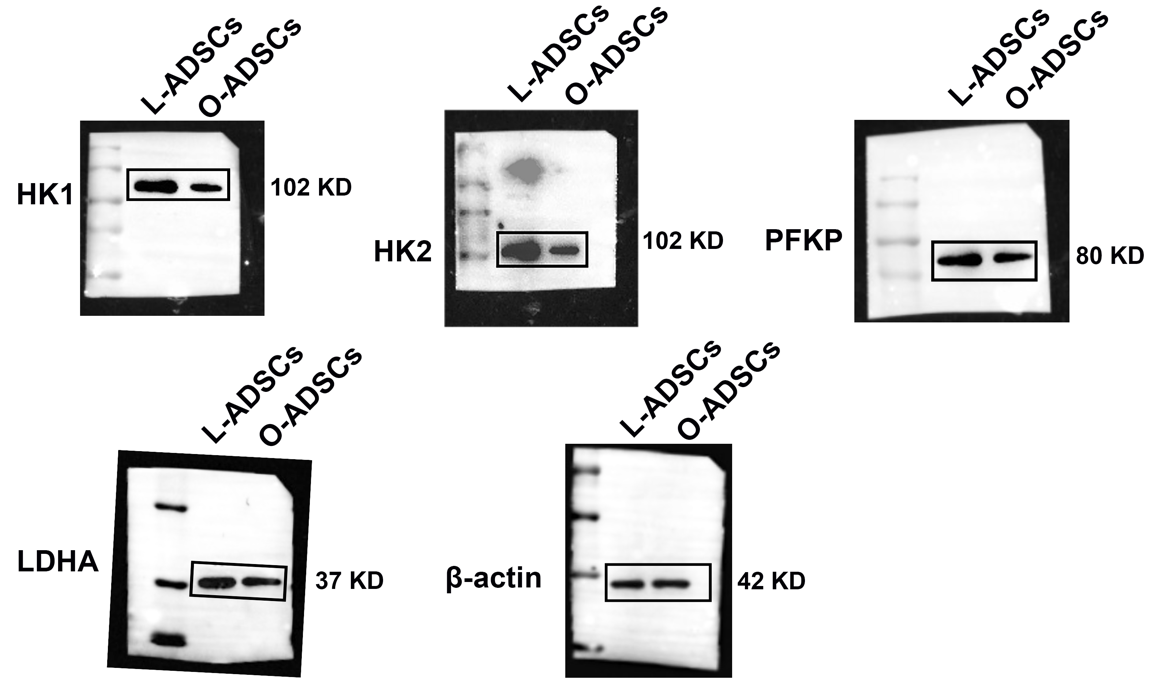


**Figure S5. Full-length blots of western blot images of figure 5G.**

**
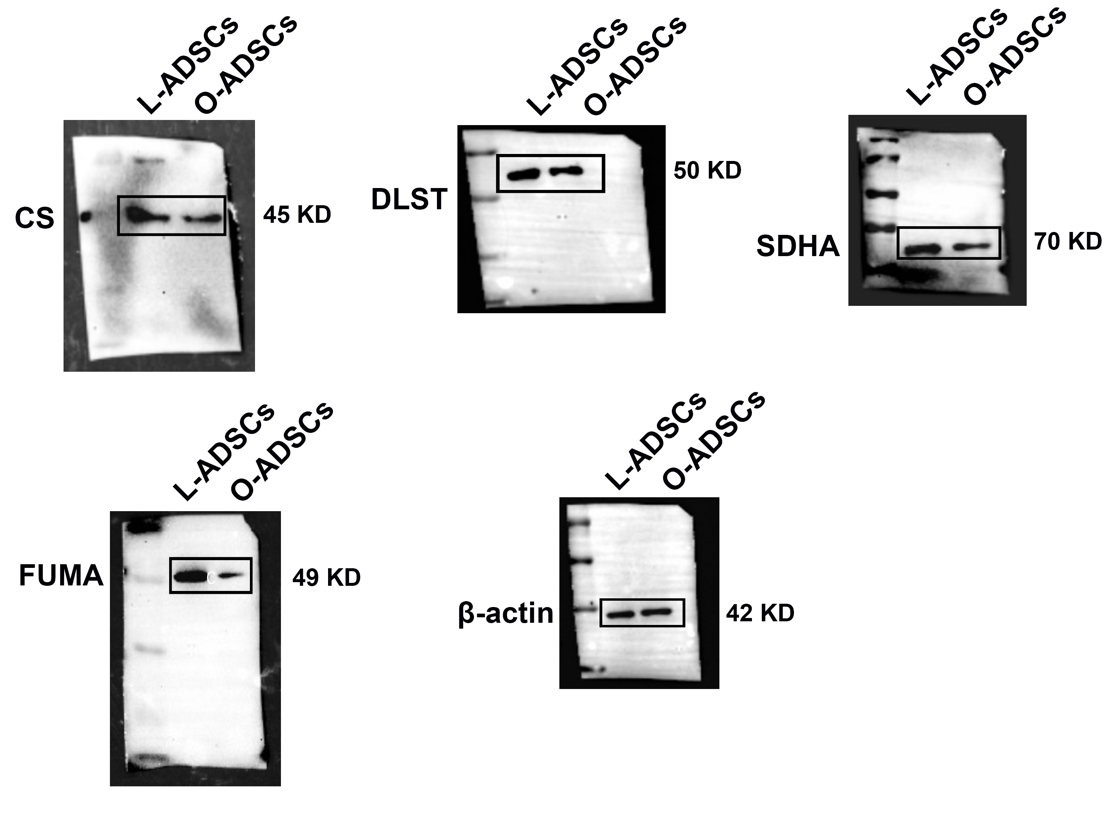
**
